# World’s First 24/7 Mobile Stroke Unit: Initial 6-Month Experience at Mercy Health in Toledo, Ohio

**DOI:** 10.3389/fneur.2018.00283

**Published:** 2018-05-17

**Authors:** Eugene Lin, Victoria Calderon, Julie Goins-Whitmore, Vibhav Bansal, Osama Zaidat

**Affiliations:** ^1^Neuroscience Institute, Mercy Health-St. Vincent Medical Center, Toledo, OH, United States; ^2^Neuroscience Institute, Mercy Health-St. Rita’s Medical Center, Lima, OH, United States

**Keywords:** cerebrovascular disease/stroke, mobile stroke unit, ambulances, telemedicine/telestroke, tissue plasminogen activator, thrombolysis

## Abstract

**Background and purpose:**

As the fourth mobile stroke unit (MSU) in the nation, and the first 24/7 unit worldwide, we review our initial experience with the Mercy Health MSU and institutional protocols implemented to facilitate rapid treatment of acute stroke patients and field triage for patients suffering other time-sensitive, acute neurologic emergencies in Lucas County, Ohio, and the greater Toledo metropolitan area.

**Methods:**

Data was prospectively collected for all patients transported and treated by the MSU during the first 6 months of service. Data was abstracted from documentation of on-scene emergency medical services (EMS) personnel, critical care nurses, and onboard physicians, who participated through telemedicine.

**Results:**

The MSU was dispatched 248 times and transported 105 patients after on-scene examination with imaging. Intravenous (IV) tissue plasminogen activator (tPA) was administered to 10 patients; 8 patients underwent successful endovascular therapy after a large vessel occlusion was identified using CT performed within the MSU without post treatment symptomatic hemorrhage. Moreover, 14 patients were treated with IV anti-epileptics for status epilepticus, and 19 patients received IV anti-hypertensive agents for malignant hypertension. MSU alarm to on-scene times and treatment times were 34.7 min (25–49) and 50.6 min (44.4–56.8), respectively.

**Conclusion:**

The world’s first 24/7 MSU has been successfully implemented with IV-tPA administration rates and times comparable to other MSUs nation-wide, while demonstrating rapid triage and treatment in the field for neurologic emergencies, including status epilepticus. With the rising number of MSUs worldwide, further data will drive standardized protocols that can be adopted nationwide by EMS.

## Introduction

Stroke has fallen from the third to the fifth leading cause of death over the past decade because of advances in prevention and treatment (1). Organizations including the American Heart Association and American Stroke Association have increased community awareness of stroke symptoms by popularizing the “FAST” and “BE-FAST” acronym. Moreover, hospitals have made stroke-care a priority by implementing policies that have significantly improved (reduced) door-to-needle times (2). There is still room for improvement, as IV-tPA can only be administered after imaging excludes a hemorrhagic stroke. The process of transporting acute stroke patients to the hospital to obtain this imaging often delays therapy by more than an hour—the equivalence of 120 million neurons dying (3).

The introduction of a mobile stroke unit (MSU), a specialized neuro-critical care ambulance with a portable CT scanner and telemedicine capabilities, circumvents this delay. They provide physicians the information and resources necessary to safely screen patients for IV-tPA eligibility and initiate thrombolytics in the field, significantly improving symptom onset to treatment times. During 2016, six MSUs were operational in U.S. metropolitan cities: Houston, Cleveland, Denver, Toledo, Memphis, and New York City, in order of launch. However, the number of MSUs continues to increase every year in the U.S. and abroad. Toledo continues to operate the only unit worldwide that is operational 24/7.

Using data from the BEnefits of Stroke Treatment Delivered Using a Mobile Stroke Unit (BEST-MSU) study, which details Houston’s implementation of its MSU, and Cleveland Clinic’s published experience from its MSU, Mercy Health introduced the world’s first 24/7 MSU in Toledo, OH, USA. In this study, we review and summarize the steps needed to establish, implement, and maintain an operational 24/7 MSU. We also present data that demonstrates the timesaving benefits our MSU has provided the community, in treating acute stroke and other neurological emergencies. We included comparisons of our patient demographics and treatment metrics to data from other MSUs. Several areas of improvements to further enhance our response and treatment times are also identified and discussed.

## Materials and Methods

Data was prospectively collected for all patients transported and treated by the MSU during the first 6 months of its operation. Patient and treatment information was documented by the Mercy Health MSU team on a standardized MSU run sheet in EPIC (Verona, WI, USA). Data was abstracted and correlated retrospectively from the run sheet and patient records. Descriptive statistical analysis was then used to organize the data (Microsoft).

### Emergency Medical Services (EMS) and Fire Department Training

Our Mercy Health MSU is located in northwest Ohio in Lucas County, which encompasses 596 square miles and has an estimated population of 433,689 (2015 U.S. Census). In addition, a bordering county to the west of the operating base is Fulton County, which covers 407 square miles and has a population of 42,537 (2015 U.S. Census).

Working with the Lucas County Emergency Medical Services (LCEMS) leadership, we elected to base our MSU near the northwest area of Lucas County. This area had the highest density of stroke diagnoses from Medicare diagnostic codes based on discharge diagnosis and patient zip codes. However, due to our MSU’s moving target radius, training was initiated with all 464 Lucas County EMS paramedic personnel, inclusive of the multiple fire departments within Lucas County. These EMS personnel had already received initial stroke training, including a 2-h interactive training with video demonstration and hands-on Rapid Arterial oCclusion Evaluation (RACE) scale assessment (2) using mock patients as a part of the RACE protocol implementation in July 2015. With adjunct training, prior to the launch of the MSU, we introduced them to the vehicle, updated scene interaction and goals, and reinforced potential stroke identification with the Cincinnati Prehospital Stroke and RACE scale. In addition, there has been ongoing training with individual squads and Toledo Fire stations since the initiation of the MSU to provide ongoing workflow improvements and feedback.

### Protocol

**Figure 1:**
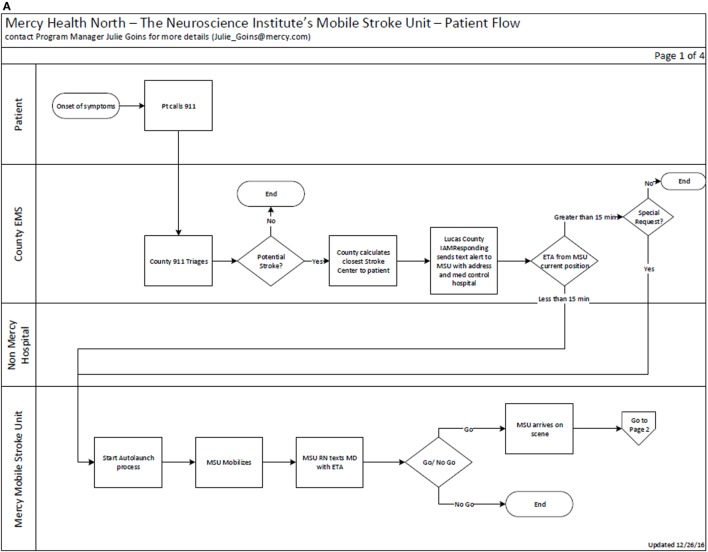

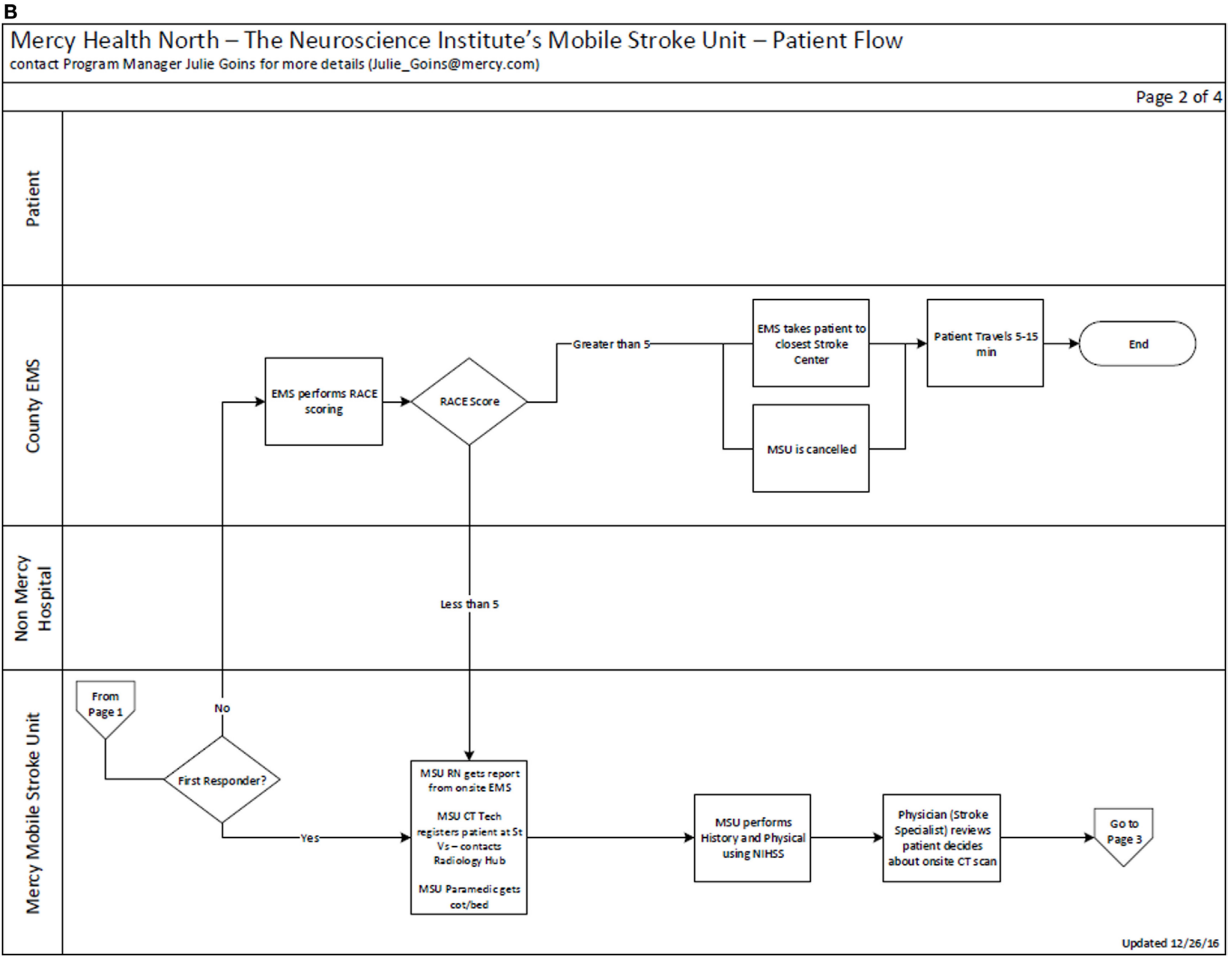
**(A)** Mercy Health Mobile Stroke Unit (MSU) workflow and triage algorithm from activation. **(B)** Mercy Health MSU activation and triage algorithm part 2.

Our MSU is alerted as a part of the LCEMS dispatch if a patient is identified as having a suspected stroke on the initial call, or upon arrival by a first responder in a 15-min radius from the MSU location (Figure 1A). The MSU and the vascular neurologist (VN) on call are notified concurrently when a run is initiated. Then every patient with possible stroke symptoms is evaluated on-scene with a focused neurological examination by our EMS personnel and critical care nurse, who are both dually trained in the NIH Stroke Scale and Emergency Neurological Life Support. If a patient is deemed to have a stroke, or was determined as a RACE candidate by the initial EMS team, a head CT is acquired (Figure 1B). During the evaluation with the MSU team, the InTouch Health Express Device is available with a VN to actively participate in performing the examination and obtain further history from the EMS team, patient, and the patient’s family.

After the on-scene neurological assessment is completed and the patient is transported to the MSU, the scan is completed and uploaded both to the cloud for access by mobile device and to our PACS system for evaluation by our neuroradiologist within 5 min. Based on the results, history, and examination, the proper course of treatment is initiated, and the patient is subsequently transported to the nearest appropriate hospital (4–6).

## Results

During the initial 6 months, our MSU transported a total of 105 patients. Another 143 dispatches were cancelled enroute to the scene after first responders evaluated the patient. The average age of our patient was 70.8 with a median of 73. When compared with initial published MSU papers from Bowry et al., Wendt et al., and Walter et al., we treated patients within a similar age range (Table 1) (3–5). In addition, we treated almost equal numbers of men and women in our first 6 months. Reviewing the risk factors for these same patients, the proportion evaluated with HTN (60%), DM (25.7%), and prior strokes (32.4%) were comparable to Charité-Universitätsmedizin MSU and University of the Saarland MSU (4, 5). Patients with a history of atrial fibrillation (18.1%) were similar to the BEST-MSU results (3).

**Table 1:** Demographics, initial diagnoses, and treatment of Mercy Health Mobile Stroke Unit (MSU) patients vs. other MSUs.

MSU (*n*)	Mercy Health MSU, Toledo (105)	University of Texas MSU, Houston—Bowry et al. (3)	Charité-Universitätsmedizin MSU, Berlin—Wendt et al. (5)	University of the Saarland MSU, Homberg—Walter et al. (4)
Age: mean ± SD	70.8 ± 15.2	64 ± UNK	73.9 ± 15.0	UNK ± UNK
Median (IQR)	73 (63–82)	UNK	UNK	72 (59–76)
Sex, men, *n* (%)	49 (46.7)	13 (50)	795 (44.1)	31 (58)
Hypertension, *n* (%)	63 (60.0)	16 (61.5)	UNK	41 (77)
Diabetes mellitus, *n* (%)	27 (25.7)	4 (15.4)	451 (25.0)	14 (26)
Atrial fibrillation, *n* (%)	19 (18.1)	5 (19.2)	440 (24.4)	12 (23)

**Initial diagnosis or treatment**				

Previous stroke	34 (32.4)	UNK	UNK	16 (30)
Intracranial hemorrhage, *n* (%)	5 (4.8)	4 (15.4)	45 (2.5)	4 (6)
Transient ischemic attack, *n* (%)	13 (12.4)	1 (3.8)	185 (10.3)	8 (15)
Patients with prehospital identification for subsequent thrombectomy, *n* (%)	8 (7.6)	4 (15.4)	UNK	UNK
Ischemic stroke, *n* (%)	43 (41.0)	11 (42.3)	610 (33.8)	29 (55)
Seizure, *n* (%)	13 (12.4)	4 (15.4)	129 (7.2)	7 (13)
Brain tumor, *n* (%)	1 (0.95)	UNK	UNK	UNK
Head trauma, *n* (%)	1 (0.95)	UNK	9 (0.5)	UNK
Uncertain initial diagnosis, *n* (%)	15 (14.3)	6 (23.1)	259 (14.4)	UNK

**Medications administered on MSU**				

**Medications, ***n***(%)**	**Frequency (105)**	**Resolution**	**Adverse events**	

Aspirin	16 (15.2)			
Lorazepam	3 (2.9)			
Nicardipine	5 (4.8)	Improvement in BP		
D 50	2 (1.9)			
Levetiracetam	14 (13.3)			
Labetalol	14 (13.3)	Improvement in BP		
Mannitol	3 (2.9)			
Clopidogrel	3 (2.9)			
tPA	10 (9.5)			
Midazolam	7 (6.7)			
Ondansetron	12 (11.4)			

An advantage to early neurological evaluation of critical neurological patients includes identification of patients who do not have ischemic strokes. Our MSU team treated a broad spectrum of patients ranging from ischemic strokes to hemorrhagic strokes and seizures (Table 1). The benefits of recognizing and managing these other neurological emergencies range from early reversal of anticoagulation related hemorrhages, to early blood pressure management after identification of subarachnoid hemorrhage, as well as the quick identification and treatment of seizure patients (Table 1). We identified 43 patients (41.0%) with likely ischemic strokes. Ten of these patients were treated with IV-tPA in the MSU. Early notification was provided to the receiving hospital of eight patients (7.6%) likely requiring endovascular treatment with mechanical thrombectomy (Table 1). We identified 5 patients (4.8%) with intracranial hemorrhage, 13 patients (12.4%) with likely seizures, 1 patient (0.95%) with brain tumor, 1 patient with head trauma (0.95%), and 15 patients with uncertain initial diagnosis (14.3%). As shown in Table 1, similar diagnoses were encountered by other MSUs.

**Figure 2:**
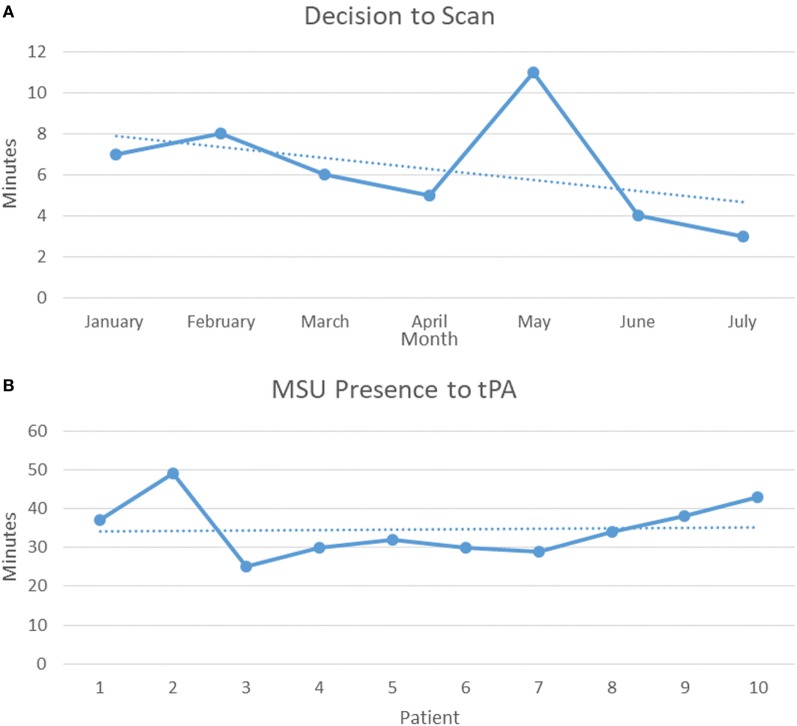
**(A)** First 6 months trend of average decision to scan time. **(B)** First 10 Mobile Stroke Unit (MSU) tPA patients from on-scene arrival to tPA administration.

**Figure 3:**
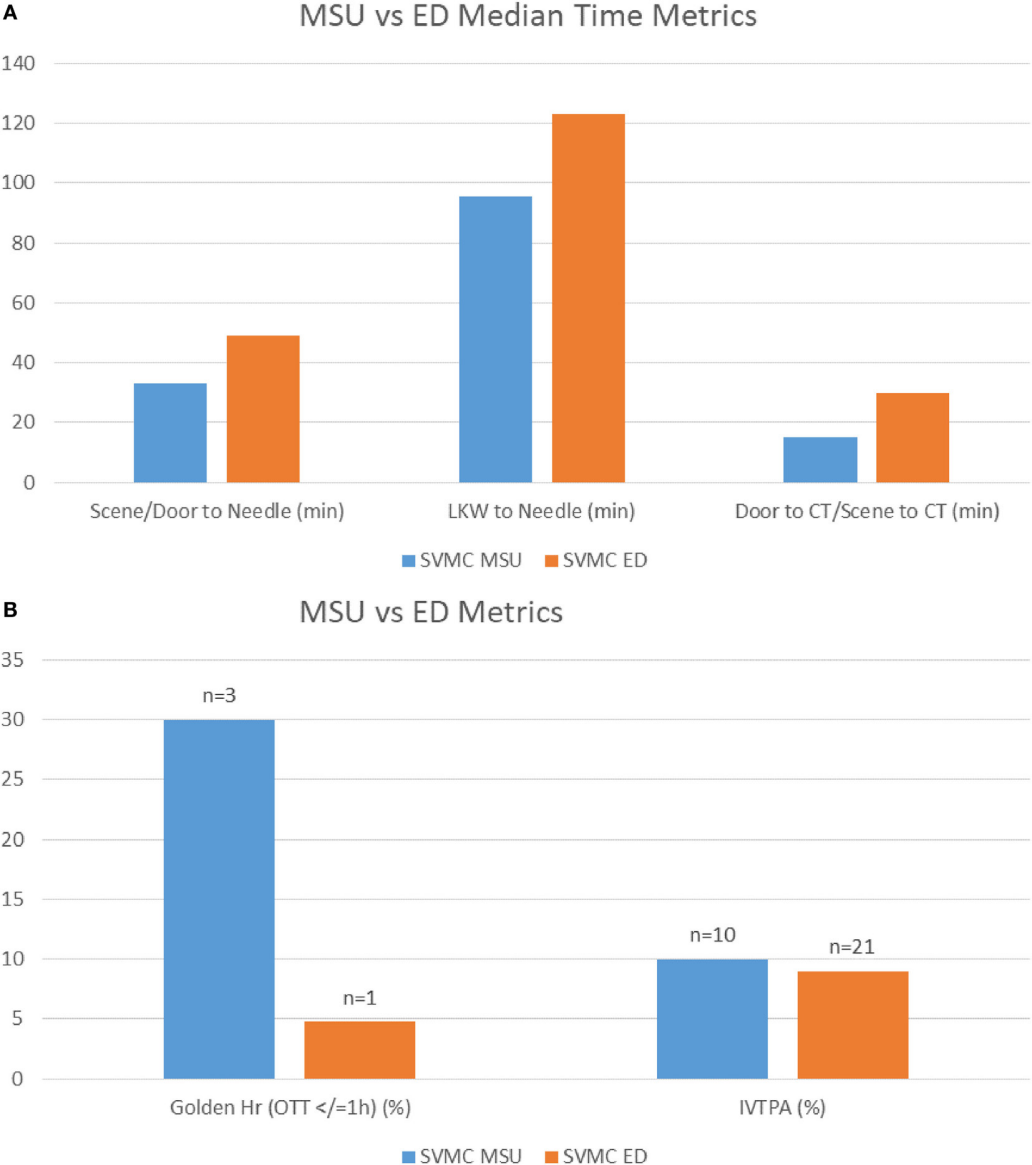
**(A)** Comparison of Mercy Health Mobile Stroke Unit (MSU) to ED patients demonstrating the difference in minutes of patient triage and stroke metrics. **(B)** Comparison of Mercy Health MSU to ED with percent of patients treated in the golden hour under 60 min and percent treated of those activated for stroke evaluation.

Finally, we demonstrated the improvement in process optimization with subsequent runs achieving faster decision to scan time in the first 180 days (Figure 2A). Furthermore, the time savings in tPA administration demonstrated in initial MSUs were supported by tPA administration times on the MSU compared to patients who presented in our ED. Median times demonstrated a decrease of 21 min when tPa was administered in the MSU vs. the ED (Figures 2B and 3A) and a corresponding decrease median time of last known well (LKW) to needle of 27.6 min on the MSU (Table 2; Figure 3A). Of those patients receiving tPA, three patients (30%) on the MSU received tPA in the golden hour (LKW), compared to one patient (4.8%) in the ED (Figures 3A,B). Our success in increasing the efficiency of tPA administration was also supported by a 50% decrease in time needed to obtain a head CT from 30 min (MSU) to 15 min (ED) (Figure 3A).

**Table 2:** Time metrics and outcomes for Mercy Health Mobile Stroke Unit (MSU), Toledo.

Rx and time metrics (*n*)	Mercy Health MSU, Toledo (*N* = 105, *n* = 10)	University of Texas MSU, Houston—Bowry et al. (3) (*N* = 26, *n* = 12)	Charité-Universitätsmedizin MSU, Berlin—Ebinger et al. (6)	University of the Saarland MSU, Homberg—Walter et al. (4)
Baseline NIH Stroke Scale		10 (3–19)		5 (3–11)
Treated with IV-tPA, *n* (%)	10 (9.5)	12 (46.2)	192 (10.6)	12 (23)
Alarm to treatment, a mean (95% CI), min^a^	50.6 (44.4–56.8)	UNK	51.8 (49.0–54.6)	UNK
Median (IQR), min	53.0 (42–59)		48 (39–56)	38 (34–42)
Last known well (LKW) to treatment, min, mean (range)	95.4 (40–153)	98 (47–265)	UNK	UNK
Median (IQR)	105 (52.0–128.8)			72 (53–108)
MSU on-scene to tPA time, min, mean (range)	34.7 (25–49)	25 (18–42)	UNK	UNK
Alarm to imaging, mean (95% CI), min	29.5 (28.0–31.0)	UNK	37.7 (35.6–39.7)	UNK [alarm to end of imaging 34 (30–38)]
Median (IQR), min	30.0 (24.3–33.3)		35 (30–42)	Median (IQR)
Alarm to INR, mean (95% CI), min	28.5 (21.6–35.4)	UNK	30.8 (28.4–33.2)	UNK
Median (IQR), min	28.5 (26.8–32.0)		26 (20–37)	
LKW to tPA < 60 min, *n* (%)	3 (30.0)	12 (33%)	UNK	UNK
Agreement on tPA eligibility between onsite and MSUVNs	UNK	90%	UNK	UNK

*^a^Treatment = IV-tPA*.

## Discussion

Every MSU program must adapt their processes and workflow to their own communities, and work closely with their local EMS and hospitals to ensure rapid care and continuity of patient management. We used the knowledge and metrics published by other MSU teams as our initial goals and further adapted them to improve and optimize treatment of critical patients. In addition to optimizing treatment of non-stroke neurological deficits, we focused on EMS identification of stroke, use of on-scene telemedicine, and pursuit of key metrics to enhance our protocols for patient care.

### Lucas County EMS and Training

The process of establishing a unified approach for rapid identification and treatment of stroke patients in the field is vital to a successful MSU. Since first responders were unfamiliar with obtaining CT imaging and administering IV-tPA in the field, follow-up training and feedback from the numerous teams that arrive on scene to evaluate and treat a patient is necessary to improve patient outcomes. Building upon the initial training conducted prior to the MSU launch, we continued to elicit feedback from our EMS teams with electronic surveys, including inquiries on on-scene interaction, appropriateness of MSU dispatch, and communication between MSU team, patient, and family. In addition, we expanded our education beyond the Lucas county paramedics by identifying the numerous fire departments that would also be involved with the on-scene triage of stroke emergencies. We developed an ongoing training plan to provide individualized feedback during their shifts and foster improved outcomes throughout our process. Using ongoing EMS, fire department, and patient feedback, we expect to reduce our on-scene times and tPA administration with improved patient treatment.

### Telemedicine

Early involvement of the physician has also supported the rapid diagnosis of patients. Protocol variation was reduced during patient evaluation with two physicians providing call coverage 24/7. During our planning phase, we elected to adopt telemedicine triage as our primary route of communication between the physician, patient, and mobile stroke team. Previous MSUs had successfully supported the benefits of telemedicine in triaging patients with low rates of technical failure and decreased process times (7, 8). We expanded our telestroke program to facilitate on-scene MSU evaluation, by adapting our existing ability to evaluate the patient through our InTouch Health (Goleta, CA, USA) mobile units. We dedicated a mobile 4G LTE hotspot to allow examination of the patient on-scene and within structures. Unlike prior MSU teams, we made it a priority to obtain concurrent evaluations with the on-scene nurse and paramedics. This allowed us to provide a more accurate assessment to determine the need for a head CT and whether a patient’s neurological examination supports further intervention and treatment. In addition, on-scene evaluations provide the ability to obtain information directly from the family or bystanders who witnessed the event and can clarify key aspects necessary for treatment, including patient LKW, baseline function, and medications. The examination proceeds once the patient is stabilized in the MSU to provide ongoing feedback to the MSU team and the assisting paramedics. Any technical concerns are addressed with direct calls to the physicians to ensure care is not delayed.

Imaging studies are obtained and uploaded both to a cloud and hospital imaging system to give image access to both the on-call evaluating VN and to the neuroradiologist. There was only one failure of image transmission to the PACS system out of 105 patients transported, and the patient was immediately taken to the nearest primary stroke center.

### Establishing Initial Metrics and Driving Improvement During the First Six Month

The need to drive an ongoing improvement feedback process, and implement that feedback, was established from the first day of service. Building upon the metrics for hospital administration of tPA, we focused on a few key treatment milestones to help increase efficacy of the MSU.

The Decision to Scan was chosen as a point of focus on the initial assessment by the physician and the MSU team, as it was the time taken to determine whether a patient would have a CT scan on the rig or be directly transported to the nearest hospital, in addition to determining whether the patient was a tPA candidate. Reviewing the initial progress during the first 6 months, we have reduced the Decision to Scan to less than 4 min by incorporating initial EMS evaluations, MSU team feedback and training, concurrent patient evaluation, and obtaining the neurological examination through on-scene nursing and physician evaluations *via* telemedicine (Figure 2A).

MSU presence to tPA is another metric we focused on to improve the efficiency of time to treatment. The impetus to improve tPA administration time has clearly been demonstrated through evaluation of tPA administration and outcome in a U.S. registry by Saver et al. The review demonstrated that in 15 min increments, faster onset to treatment time was associated with decreased hospital mortality, reduced symptomatic hemorrhage, and increased achievement of independent ambulation at discharge (9). Improved outcomes from faster tPA administrator was supported in a recent study that analyzed the benefits of reducing door-to-needle times; median delivery decreased from 77 to 67 min (10). We dedicated training and focus on concurrent actions for our nurses and team members to reduce the time to administration, once the decision to give tPA has been made. Our results support more streamlined administration of tPA over time, comparable to other MSUs (Table 2) and our ED times (Figures 3A,B). Exposure across all team members and different shifts resulted in a median 33 min tPA administration time through our 6-month period from MSU presence to IV-tPA (Table 2; Figures 2B and 3A).

### Treatment Is Not Limited to tPA

In addition to providing a comprehensive evaluation to patients 24/7, we have worked with our MSU team to expand the scope beyond IV-tPA administration for ischemic stroke or a decision pathway for mechanical thrombectomy. Over the past 6 months, our data demonstrated that our MSU can evaluate and treat a range of other neurological emergencies (Table 1). The spectrum of pathologies identified and treated includes seizures, complex migraine headaches with aura, hemorrhages, tumors, and hypoglycemia. The MSU’s ability to make these critical decisions also improves the appropriate use of hospital resources to identify patients who may benefit from further triage at a comprehensive stroke center, as well as triage non-stroke and less critical patients to the the nearest hospital (5), as demonstrated with other MSUs (5).

## Conclusion

Operating a 24/7 MSU provides early evaluation and treatment of stroke and and other acute, neurologic emergencies. Similar to data compiled from other MSUs, IV-tPA candidates received therapy under an hour of LKW without increased complication rates. Moreover, constant feedback from our local EMS allowed us to revise our protocols and ensure more efficient patient care. For example, we have implemented the RACE score to triage patients for potential thrombolytics. There were no major issues related to the unit’s full-time operation.

In addition to providing earlier therapy with thrombolytics for acute stroke patients, patients with hypertensive cerebral hemorrhage, status epilepticus, and/or severe vasogenic edema can receive rapid treatment. Early initiation of these treatments is important as patients with hypertensive hemorrhage are at highest risk for rehemorrhage during the first 6–8 h. A significant predictor being refractory severe hypertension. The ability to rapidly diagnose the hemorrhage using the mobile CT unit allows for early aggressive treatment of elevated blood pressure. Moreover, it is well known that prolonged status epilepticus significantly worsens prognosis for survival. Thus, having the MSU equipped with IV anti-epileptics allows for earlier resolution of status epilepticus and improved prognosis.

With the knowledge obtained through our first 6 months of 24/7 services, we expect further improvements in time metrics and increases in the number of patients who will benefit from faster treatment of acute neurological emergencies in the field.

## Author Contributions

EL, VC, JG-W, VB, and OZ designed the study, developed the methodology, collected and analyzed the data, wrote and edited the manuscript, and gave final approval.

## Conflict of Interest Statement

The authors declare that the research was conducted in the absence of any commercial or financial relationships that could be construed as a potential conflict of interest.
